# Air pollution exposure may impact the composition of human milk oligosaccharides

**DOI:** 10.1038/s41598-024-57158-z

**Published:** 2024-03-20

**Authors:** Noopur C. Naik, Elizabeth A. Holzhausen, Bridget N. Chalifour, Maria M. Coffman, Fredrick Lurmann, Michael I. Goran, Lars Bode, Tanya L. Alderete

**Affiliations:** 1 Department of Integrative Physiology, University of Colorado Boulder, Boulder, CO USA; 2 Cleveland Clinic Lerner College of Medicine at Case Western Reserve University College of Medicine, Cleveland, OH USA; 3 Sonoma Technology, Inc., Petaluma, CA USA; 4 Department of Pediatrics, Children’s Hospital of Los Angeles, Los Angeles, CA USA; 5 Department of Pediatrics, Larson-Rosenquist Foundation Mother-Milk-Infant Center of Research Excellence (MOMI CORE), Human Milk Institute (HMI), University of California, San Diego, La Jolla, CA USA

**Keywords:** Air pollution, Human milk oligosaccharides, Early life, Infant development, Environmental exposure, Physiology, Environmental impact

## Abstract

Human milk oligosaccharides (HMOs) impact neonate immunity and health outcomes. However, the environmental factors influencing HMO composition remain understudied. This study examined the associations between ambient air pollutant (AAP) exposure and HMOs at 1-month postpartum. Human milk samples were collected at 1-month postpartum (n = 185). AAP (PM_2.5_, PM_10_, NO_2_) exposure included the 9-month pregnancy period through 1-month postpartum. Associations between AAP with (1) HMO diversity, (2) the sum of sialylated and fucosylated HMOs, (3) 6 a priori HMOs linked with infant health, and (4) all HMOs were examined using multivariable linear regression and principal component analysis (PCA). Exposure to AAP was associated with lower HMO diversity. PM_2.5_ and PM_10_ exposure was positively associated with the HMO 3-fucosyllactose (3FL); PM_2.5_ exposure was positively associated with the sum of total HMOs, sum of fucosylated HMOs, and the HMO 2′-fucosyllactose (2′FL). PCA indicated the PM_2.5_, PM_10_, and NO_2_ exposures were associated with HMO profiles. Individual models indicated that AAP exposure was associated with five additional HMOs (LNFP I, LNFP II, DFLNT, LNH). This is the first study to demonstrate associations between AAP and breast milk HMOs. Future longitudinal studies will help determine the long-term impact of AAP on human milk composition.

## Introduction

Human milk plays an important role in infant nutrition and overall well-being, offering protection against numerous infections and diseases such as colitis, infantile diarrhea, diabetes, and obesity^1–3^. It is enriched with specific bioactive compounds that are instrumental in infant growth and developmental processes^4–6^. Notably, human milk oligosaccharides (HMOs), which rank as the third most abundant component in human milk, comprise over 150 distinct oligosaccharides^7,8^.

HMOs, which are synthesized from five core monosaccharides^9–11^, serve as prebiotics for the developing gut microbiome^12^, protect against infections^13^, stimulate neurological development^14^, and act as anti-inflammatory and immune modulators^15,16^. Moreover, a reduced HMO diversity has been linked to adverse infant health outcomes, such as necrotizing enterocolitis (NEC)^17,18^. Experimental investigations have highlighted specific HMOs, such as DSLNT, for their protective attributes against NEC^18^. One study^19^ highlighted the potential of 2’FL and 3FL in modulating immunity, as they bind to adhesion molecules pivotal for dendritic cell activation and emulate cell surface receptors, offering protection against infection^16^. Additionally, research has shown that LNT and 6’SL can activate GPCR35, a mediator in alleviating pain and colitis symptoms^20^. Collectively, these findings underscore the integral role of various HMOs in promoting infant health.

The composition and concentration of HMOs varies greatly among women^21^ and across the time course of lactation^22^ Variations in HMO concentrations have been linked with genetic secretor status^9,23^, geographic location^9,10,24^, socioeconomic status^25^, pre-pregnancy body mass index (BMI)^26^, maternal age^26^, ethnicity^9^, obesity^27^, and lactation period^9,22^. Beyond these factors, exposure to air pollutants may also impact human milk composition. Exposure to air pollution varies by geographic location^28^ and airborne pollutant concentrations have been mitigated by vegetation and green space^29^. Accordingly, geography has been linked to differences in HMO profiles^9,30^, as have residential green environments^31^. Air pollution exposure has also been found to impact human milk composition, including lipid biosynthesis^32^. Given the genetic underpinnings of HMO biosynthesis, exposure to air pollution might modulate epigenetic pathways^33,34^, subsequently influencing HMO production within mammary glands.

The primary aim of this study was to therefore determine whether maternal AAP exposure was associated with summary measures of HMOs (i.e., diversity, sum of sialylated and fucosylated) as well as concentrations of six HMOs that have been previously linked with infant health (i.e., 2’FL, 3FL, 6’SL, LNnT, LNT, DSLNT). Based on existing literature, it was hypothesized that higher exposure to AAP would be associated with lower HMO diversity and the concentrations of six HMOs that have previously been found to impact infant health^12,16,19,20,35–37^. As a secondary aim, the associations between AAP and HMOs were examined using principal component analysis (PCA) as a data reduction approach. Lastly, as an exploratory and hypothesis generating analysis, we examined the associations between AAP with all 19 HMOs our analytical platform can quantify with confidence. Furthermore, this study follows a cohort of Latino and Hispanic women. Hispanic and Latino individuals have a greater risk of developing type 2 diabetes^38^ and obesity^39^, but are underrepresented in biomedical research^40^. Results from this study have the potential to uncover potentially important associations between AAP and human milk composition in an unstudied population of Latino and Hispanic postpartum women.

## Results

### Study population characteristics

**Figure 1:**
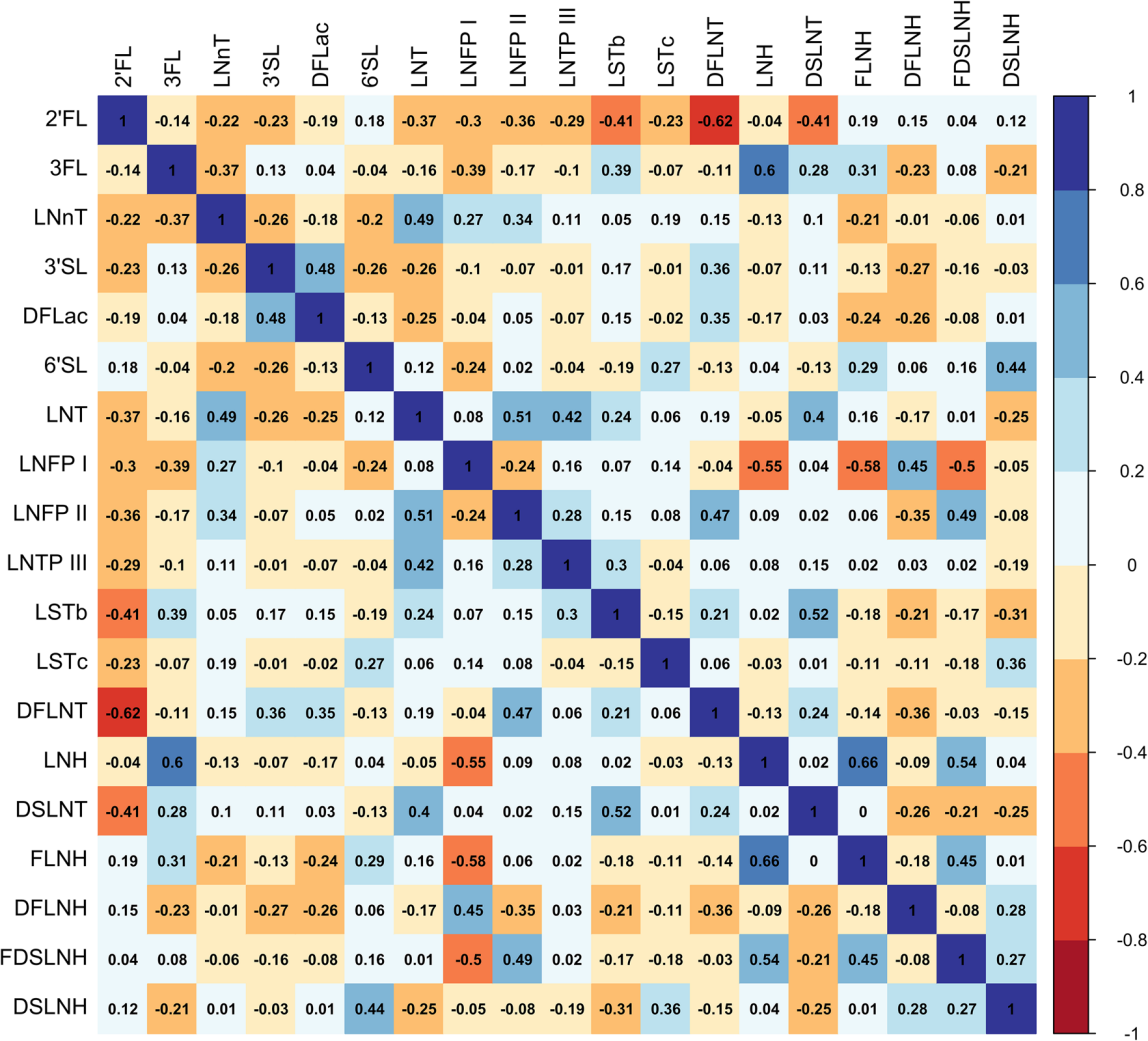
Correlations among HMOs examined in the Mother’s Milk Study. Correlation matrix among HMOs (nmol/mL) using Pearson correlation coefficients. The color of each square, either red (negative) or blue (positive), denotes the direction of the correlation.

Table 1 displays the mean physical, social, and environmental characteristics of the 185 mothers included in the analysis. On average, mothers were 29 years of age (range: 18–45 years), and overweight before pregnancy (BMI: 28.68 ± 6.03 kg/m^2^). Additionally, 28.6% of mothers were healthy weight, 32.4% were overweight, and 38.9% were obese. All mothers in the sample were in the mature milk stage where, on average, breastmilk samples were collected 32.5 days after delivery. The Hollingshead Index was used to measure the participant’s socioeconomic status, where 51.9% had low or very low SES (Hollingshead score below 26.5). Maternal exposure to PM_2.5_, PM_10_, and NO_2_ were moderately to highly correlated. As shown in Table 2, NO_2_ was moderately correlated with PM_10_ (r = 0.61, *p* < 2.2e-16) and PM_2.5_ (r = 0.65, *p* < 2.2e-16). Additionally, PM_10_ and PM_2.5_ were highly correlated (r = 0.85, *p* < 2.2e-16). When examining HMOs, we observed that the strength of the Pearson correlation coefficients among the HMOs varied and was contingent upon the specific HMOs being examined **(**Fig. 1**)**. Lastly, we examined correlations between maternal factors and HMOs, finding that maternal age and BMI were only weakly correlated with HMO concentrations (Supplemental Fig. 1).

**Table 1:** Descriptive statistics.

Variable	Mean ± SD
Maternal characteristics	
Pre-pregnancy BMI (kg/${m}^{2}$)	28.68 ± 6.03
Age (years)	28.99 ± 6.22
Breast milk stage (days after delivery)	32.47 ± 4.11
SES status (hollingshead index)	26.24 ± 11.78
Maternal AAP exposure*	
PM_2.5_ (µg/${m}^{3}$)	11.89 ± 1.02
PM_10_ (µg/${m}^{3}$)	29.97 ± 3.65
NO_2_ (ppb)	17.99 ± 2.23

Table shows the mean ± standard deviation (SD) for 185 mothers included in this analysis. Units for all values are indicated. * Maternal AAP exposure is shown. Briefly, air pollution exposures were calculated using a weighted average of exposure during the pregnancy period (281 days) through the postpartum period defined as $(\frac{Pregnancy\, Period}{Pregnancy \,Period\,+\,Days\, Postpartum\, at\, Baseline}*Pregnancy\, Exposure)+(\frac{Days\, Postpartum\, at\, Baseline}{Pregnancy\, Period\,+\,Days\, Postpartum\, at\, Baseline}*Postpartum\, Exposure)$.

**Table 2:** Correlations among maternal ambient air pollutant exposures.

	NO_2_	PM_10_	PM_2.5_
NO_2_	–	0.61*	0.65*
PM_10_	0.61*	–	0.85*
PM_2.5_	0.65*	0.85*	–

Correlation matrix among maternal ambient air pollutant exposures using Pearson’s correlation coefficient (r). All correlations were statistically significant with *p < 2.2e-16.

### Ambient air pollution exposure was associated with HMO composition

**Figure 2:**
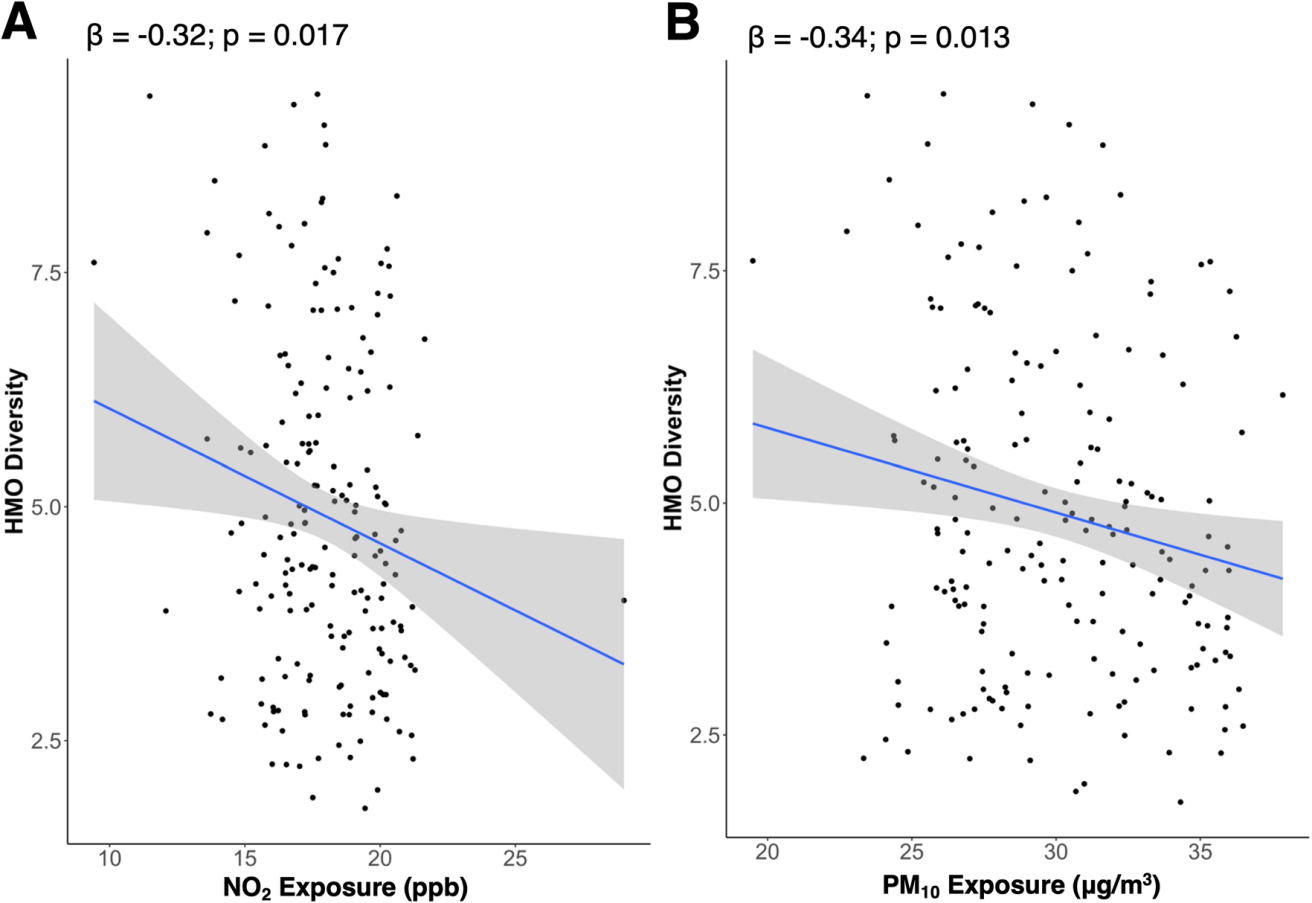
Higher exposure to NO_2_ and PM_10_ was significantly associated with lower and HMO diversity. Figures show unadjusted data with the effect estimates (β) and unadjusted *p* values from the multivariate linear regression models for NO_2_ (**A**) and PM_10_ (**B**). Multivariable models adjusted for maternal age and socioeconomic status. Effect estimates (β) are reported for a one standard deviation increase in NO_2_ (SD: 2.23 ppb) and PM_10_ (SD: 3.65 µg/${m}^{3}$) exposure.

**Figure 3:**
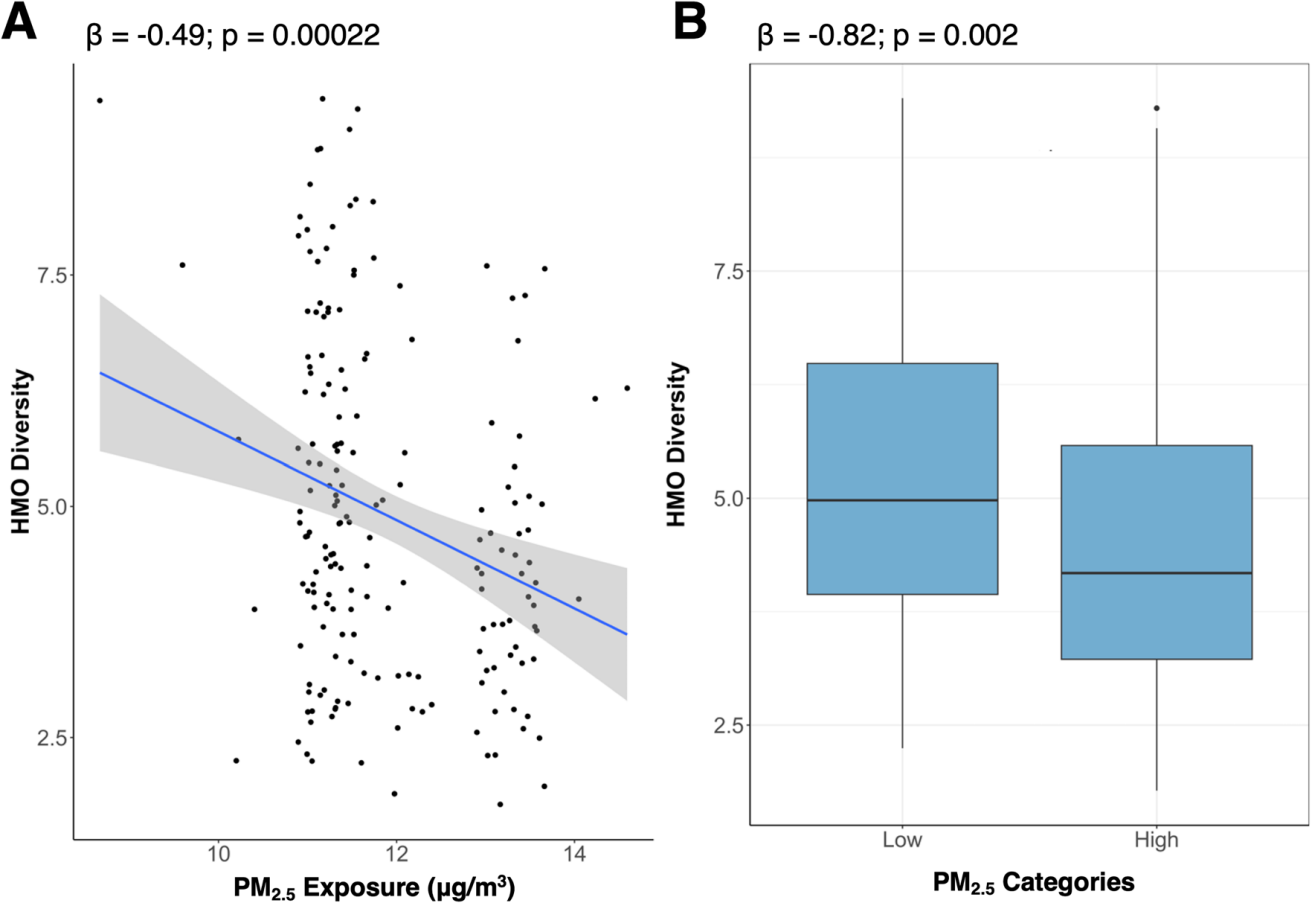
Higher exposure PM_2.5_ was associated with lower HMO diversity. (**A**) Figure shows the associations between PM_2.5_ exposure and HMO diversity. (**A**) Effect estimates (β) and unadjusted *p* values from the multivariate linear regression models are shown on the unadjusted plot. The effect estimate (β) is reported for a one standard deviation increase in PM_2.5_ exposure (SD: 1.02 µg/${m}^{3}$). (**B**) Boxplots for HMO diversity in the high and lower PM_2.5_ exposure groups. Effect estimates (β) and unadjusted *p* values from the multivariate linear regression models are shown on the unadjusted boxplot. All models adjusted for maternal age and socioeconomic status.

As shown in Table 3, breastmilk HMOs at 1-month post-partum were associated with maternal exposure to AAP. For example, a 1-SD higher NO_2_, PM_10_, and PM_2.5_ was associated with a 0.32 (*p* = 0.02), 0.34 (*p* = 0.01) and 0.49 lower HMO diversity (*p* < 0.001), respectively **(**Table 3 and Fig. 2**).** Further, NO_2_ was negatively associated with the sum of sialylated HMOs (β = − 114.53, *p* = 0.04). PM_10_ exposure was positively associated with 3FL, where each 1-SD increase in PM_10_ exposure was associated with a 239.38 nmol/mL higher 3FL (*p* = 0.02). Additionally, a 1-SD higher PM_2.5_ exposure was positively associated with the total sum of all HMOs (β = 398.96, *p* = 0.002) and the sum of fucosylated HMOs (β = 287.84, *p* = 0.04), as well as human breast milk concentrations of 2’FL (β = 558.66, *p* = 0.01) and 3FL (β = 336.68, *p* < 0.001). In the current study, PM_2.5_ had a bimodal distribution. Therefore, PM_2.5_ was also examined as a categorical variable based on a median split (high/low) where results remained largely consistent with those observed when examining PM_2.5_ as a continuous exposure (Table 4). For example, increased exposure to PM_2.5_ was associated with lower HMO diversity when treated as a continuous (*p* < 0.001) and as a categorial exposure (*p* = 0.002) (Fig. 3A,B). However, the sum of fucosylated HMOs was no longer significantly associated with PM_2.5_ exposure when examined as a dichotomous variable (Table 4).

**Table 3:** Ambient air pollution exposure was associated with a priori HMOs.

	NO_2_		PM_10_		PM_2.5_	
	β	*P* Value	β	*P* Value	β	*P* Value
HMO summary measures						
Diversity	− 0.32	**0.02**	− 0.34	**0.01**	− 0.49	**0.0002**
Sum (nmol/mL)	71.98	0.58	177.46	0.17	398.96	**0.002**
HMO-bound sialic acid (nmol/mL)	− 114.53	**0.04**	− 61.79	0.28	− 60.40	0.28
HMO-bound fucose (nmol/mL)	132.87	0.34	115.37	0.41	287.84	**0.04**
HMO concentrations						
2’FL (nmol/mL)	250.83	0.27	251.06	0.27	558.66	**0.01**
6’SL (nmol/mL)	1.18	0.96	38.53	0.11	27.64	0.25
DSLNT (nmol/mL)	− 19.31	0.18	− 12.45	0.39	− 12.72	0.37
3FL (nmol/mL)	35.84	0.72	239.38	**0.02**	336.68	**0.0008**
LNT (nmol/mL)	− 75.40	0.09	− 41.95	0.36	− 41.18	0.36
LNnT (nmol/mL)	2.56	0.91	− 12.66	0.58	− 12.78	0.57

Effect estimates (β) and unadjusted *p* values from the multivariate linear regression models are shown. Models adjusted for maternal age and socioeconomic status. All effect estimates (β) are reported for a one standard deviation increase in NO_2_ (SD: 2.23 ppb), PM_10_ (SD: 3.65 µg/${m}^{3}$), and PM_2.5_ exposure (SD: 1.02 µg/${m}^{3}$). Unadjusted *P*-values less than 0.05 are denoted in bold text.

**Table 4:** High PM_2.5_ exposure was associated with a priori HMOs.

	β	*P* Value
HMO summary measures		
Diversity	− 0.82	**0.002**
Sum (nmol/mL)	788.70	**0.002**
HMO-bound sialic acid (nmol/mL)	24.82	0.83
HMO-bound fucose (nmol/mL)	485.25	0.08
HMO concentrations		
2’FL (nmol/mL)	899.63	**0.05**
6’SL (nmol/mL)	61.71	0.20
DSLNT (nmol/mL)	3.75	0.89
3FL (nmol/mL)	763.23	**0.0001**
LNT (nmol/mL)	− 48.53	0.59
LNnT (nmol/mL)	− 53.24	0.24

Effect estimates (β) and unadjusted *p* values from the multivariate linear regression models are shown where PM_2.5_ was treated as a categorical variable (high/low). Multivariable models adjusted for maternal age and socioeconomic status. Unadjusted *P*-values less than 0.05 are denoted in bold text.

### Ambient air pollution exposure was associated HMO PCs

A principal components (PC) analysis was performed on all 19 HMOs as a data reduction technique to further examine the associations between AAP exposures and HMOs. The eigenvalues for the top six PCs, which explain ~ 72% of the total variance in the data, are shown in Table 5. Eigenvalues were further explored if they met a threshold ≥ 1. The loadings of each HMO against PC1 (x-axis) and PC2 (y-axis), where 2’FL, DFLNT, LSTb, DSLNT, LNT, and FLNH contributed to PC1 and LNFPI, LNH, FLNH, 3FL, FDSLNH, and DFLNH contributed to PC2 (Supplemental Fig. 2). PCs 1 to 6 were analyzed with respect to AAP after adjusting for maternal age and socioeconomic status (Table 6). Briefly, PM_2.5_ was negatively associated with PC1 (*p* = 0.009), while NO_2_ was positively associated with PC2 (*p* = 0.05), and PM_10_ (*p* = 0.01) and PM_2.5_ (*p* = 0.005) were positively associated with PC4. Given these findings, PC1, PC2, and PC4 were then further analyzed to determine which HMOs contributed most to their profiles. This was done by ranking the magnitude of the loading scores of the HMOs in the profiles (Table 7). The HMOs with the highest contribution to PC1 were 2’FL, DFLNT, LSTb, DSLNT, LNT, and FLNH. The HMOs with the highest contribution to PC2 were LNFPI, LNH, FLNH, 3FL, FDSLNH, and DFLNH. The HMOs with the highest contribution to PC4 were DSLNH, DFLac, DFLNT, LSTc, LNFP II, and 3’SL. Of these HMOs, four (i.e., 2’FL, DSLNT, 3FL, and LNT) overlapped with those that we explored as part of our a priori analysis.

**Table 5:** Eigenvalues and aggregate proportion of variance explained by principal components.

Principal component	Eigenvalue	Aggregate proportion of variance explained (%)
PC1	1.88	18.66
PC2	1.79	35.46
PC3	1.58	48.65
PC4	1.39	58.83
PC5	1.21	66.50
PC6	1.07	72.53

Eigenvalues of principal components (PCs) 1–6 that were all above a value of one, indicating strength of the PC. Aggregate proportion of variance explained by including each additional PC is also shown.

**Table 6:** Exposure to ambient air pollution was associated with HMOs based on a principal component analysis.

	NO_2_		PM_10_		PM_2.5_	
	β	*P* Value	β	*P* Value	β	*P* Value
PC1	− 0.17	0.23	− 0.26	0.06	− 0.36	**0.009**
PC2	0.26	**0.05**	− 0.05	0.73	− 0.18	0.19
PC3	− 0.13	0.27	− 0.08	0.49	− 0.13	0.26
PC4	0.18	0.09	0.26	**0.01**	0.29	**0.005**
PC5	− 0.03	0.75	0.11	0.25	0.05	0.59
PC6	0.04	0.62	0.04	0.65	0.13	0.10

Shows the results from the multivariate linear regression analysis with ambient air pollutants as the independent variables and PC1 through PC6 as the dependent variables. Models adjusted for maternal age and socioeconomic status. All effect estimates (β) are reported for a one standard deviation increase in NO_2_ (SD: 2.23 ppb), PM_10_ (SD: 3.65 µg/${m}^{3}$), and PM_2.5_ exposure (SD: 1.02 µg/${m}^{3}$). Unadjusted *P*-values less than 0.05 are denoted in bold text.

**Table 7:** HMO loading scores for PC1, PC2, and PC4.

Loading Scores					
PC1		PC2		PC4	
2’FL (nmol/mL)	− 0.39	LNFP I (nmol/mL)	0.42	DSLNH (nmol/mL)	− 0.37
DFLNT (nmol/mL)	0.34	LNH (nmol/mL)	− 0.42	DFLac (nmol/mL)	− 0.36
LSTb (nmol/mL)	0.32	FLNH (nmol/mL)	− 0.38	DFLNT (nmol/mL)	− 0.35
DSLNT (nmol/mL)	0.29	3FL (nmol/mL)	− 0.33	LSTc (nmol/mL)	− 0.29
LNT (nmol/mL)	0.27	FDSLNH (nmol/mL)	− 0.33	LNFP II (nmol/mL)	− 0.27
FLNH (nmol/mL)	− 0.23	DFLNH (nmol/mL)	0.31	3’SL (nmol/mL)	− 0.26

Shows the loading scores for the HMOs that contributed most to PC1, PC2, and PC4. A higher magnitude indicates a greater contribution to the PC.

### Exploratory analysis identified that ambient air pollution exposure was associated with five additional HMOs

As an exploratory analysis, the relationship between AAP exposure with HMOs was examined (Table 8). Again, a multivariate regression analysis was performed that adjusted for maternal age and socioeconomic status. While none of these associations survived correction for multiple testing (P_FDR_ > 0.05), adjusted *p*-values indicated that AAP exposure was associated with several HMOs. Specifically, AAP was associated with five HMOs, including a negative association with LNFP I, LNFP II, and DFLNT, and a positive association with LNH and FLNH (unadjusted *p* < 0.05).

**Table 8:** Ambient air pollution exposure was associated with breastmilk HMOs.

	NO_2_			PM_10_			PM_2.5_		
	β	*P* Value	P_FDR_	β	*P* Value	P_FDR_	β	*P* Value	P_FDR_
LNFP I (nmol/mL)	105.47	0.23	0.43	− 36.10	0.68	0.74	− 178.41	**0.041**	0.22
LNFP II (nmol/mL)	− 83.34	**0.0045**	0.17	− 68.33	**0.021**	0.17	− 70.27	**0.017**	0.17
DFLNT (nmol/mL)	− 74.56	0.18	0.38	− 119.02	**0.032**	0.21	− 145.13	**0.008**	0.17
LNH (nmol/mL)	− 2.42	0.61	0.69	6.43	0.18	0.38	9.38	**0.047**	0.22
FLNH (nmol/mL)	− 8.28	0.33	0.52	14.58	0.09	0.24	19.47	**0.021**	0.17

Multivariable models adjusted for maternal age and socioeconomic status. Unadjusted *P*-values less than 0.05 are denoted in bold text. The table also includes the adjusted (P_FDR_) *p*-values from the exploratory analysis. All effect estimates (β) are reported for a one standard deviation increase in NO_2_ (SD: 2.23 ppb), PM_10_ (SD: 3.65 µg/${m}^{3}$), and PM_2.5_ exposure (SD: 1.02 µg/${m}^{3}$).

## Discussion

To our knowledge, this is the first study to examine the associations between AAP and HMO composition. Increasing evidence suggests that HMOs may impact infant health, including establishing the infant gut microbiome, modulating host-epithelial immune responses, and stimulating neurological development^15,41^. Previous research also suggests that HMO composition varies significantly across mothers due to factors that are not fully understood but do appear to include genetics^23^ and geography^9,10,24^. Therefore, the current study sought to determine if exposure to AAP was associated with breastmilk HMOs among 185 Latino mothers at 1-month post-partum. Using three analytical approaches, we found that exposure to AAP was associated with lower HMO diversity as well as an increased sum of HMOs, sum of sialylated HMOs, and abundance of concentrations of 2′FL and 3FL. These results provide the first evidence that exposure to air pollutants may impact the composition of human breast milk.

Results from this study indicate that higher exposure to AAP was associated with lower HMO diversity, which has been linked to adverse health outcomes in infants, including poor immunity and increased susceptibility to infections^25^. Additionally, higher HMO diversity has been associated with lower fat mass in infants^42^. A previous study found that HMO diversity differed among women from different geographic locations^10^, suggesting that exposure to air pollutants may partially explain these geographic differences. Another study^31^ found that increased green space was correlated with higher HMO diversity. As green space can mitigate air pollutant concentrations^29^, our findings of an inverse relationship between HMO diversity and air pollution exposure are consistent with this study. In addition to examining summary measures of HMOs, we also explored six a priori HMOs that have been linked with infant growth and development. Among these HMOs, PM_10_ exposure was positively associated with 3FL, and PM_2.5_ was positively associated with both 2′FL and 3FL. 2′FL and 3FL have been linked to infant immunity, with 2’FL being protective against necrotising enterocolitis by inhibiting TLR-4^37^ and 3FL binding adhesion molecules to impact immunity^19^. However, the protective effects of specific HMOs may vary based on the overall composition of breastmilk, the infant gut microbiota, maternal and infant genetics, or the health status of the infant. Further, previous studies have found that breastfeeding–possibly due to HMO composition–has a protective effect against some of the adverse health effects of air pollution on the respiratory and immune systems^43^. Therefore, it is also possible that this effect is partly due to HMO mitigation, where women exposed to higher levels of air pollution may produce more of these HMOs to provide infants with enhanced immunity.

In addition to examining six a priori HMOs, principal component (PC) analysis was performed as a data reduction method. Based on this approach, each PC can be interpreted as a unique HMO “profile”, in which different HMOs have varying levels of importance for a specific profile based on their loading score. AAP exposure was associated with the profiles contained with PC1, PC2, and PC4. For example, PM_2.5_ exposure was associated with PC1 and PC4, PM_10_ exposure was also associated with PC4, and NO_2_ exposure was associated with PC2. Based on the loading scores, we observed that PC1 was largely defined by 2′FL, DFLNT, LSTb, DSLNT, LNT, and FLNH, PC2 was largely defined by LNFPI, LNH, FLNH, 3FL, FDSLNH, and DFLNH, and PC4 was largely defined by DSLNH, DFLac, DFLNT, LSTc, LNFP II, and 3′SL. Of these HMOs, four (i.e., 2′FL, 3FL, DSLNT, LNT) overlapped with our six a priori HMOs.

Lastly, we took an agnostic approach and examined the individual associations between each air pollutant and the HMOs we hadn’t included in our a priori HMO analysis. Using this approach, we identified an additional five HMOs beyond 2’FL and 3FL that were associated with one or more air pollutant. For example, PM_2.5_ exposure was associated with a lower LNFP I concentration and higher LNH concentration. Both LNFP I and LNH have been linked with the composition of the infant gut microbiota^44^, including an higher abundance of *Staphylococcus*^44^. Because air pollution exposure may also impact the gut microbiome^45–47^, additional research into these associations would help elucidate the relationship between air pollution, HMOs, and the gut microbiome. Furthermore, exposure to NO_2_, PM_10_, and PM_2.5_ was inversely associated LNFP II. This is important since higher concentrations of LNFP II have been associated with fewer respiratory problems in infants^48^. Hence, mothers exposed to higher levels of air pollution may also produce lower levels of LNFP II, which could place their infants at an increased risk for respiratory issues due to their existing higher air pollution exposures and lower LNFP II intake. Lastly, we found that FLNH was positively associated with PM_2.5_ and DFLNT was inversely associated with both PM_10_ and PM_2.5_ exposures, although the health implications of these HMOs are largely unknown.

Previous work suggests that exposure to air pollution impacts breastmilk composition. For example, an experimental study demonstrated that rabbits exposed to diesel particulate matter during pregnancy led to the development of fat globules in the mammary glands as well as increased expression of genes for enzymes involved in lipid biosynthesis^32^. Due to the importance of genetic factors in synthesizing HMOs, there is biological plausibility that exposure to air pollution may impact HMO production and composition. Emerging data suggests that air pollution exposure modulates DNA methylation throughout the lifespan^33^. Additionally, during pregnancy there are DNA methylation changes that occur in mammary epithelial cells^49^ where studies suggest that epigenetics may play an role in mammary gland development and function^50^. Additionally, exposure to air pollution has been linked with circulating miRNAs^51,52^, which may alter gene expression and HMO composition via impacts on the mammary gland^53,54^. Given that there is a genetic component of HMO biosynthesis^55^, and HMOs are solely made in the mammary gland, evaluating the effect of air pollution on HMO composition warrants further research.

While this is the first study to examine the relationship between AAP and HMO composition, there are several limitations which should be considered. This study was cross-sectional, making it difficult to establish a causal relationship between air pollution and HMO composition. Additionally, air pollution estimates were generated using individual residential address histories, so misclassification of exposures may have occurred due to time spent away from the provided addresses, or sources of indoor air pollution^56^. The inverse distance-squared weighting (IDW2) exposure assessment method used is also limited by spatial resolution compared to other exposure assessment methods such as land-use-regression or hybrid models^57^. However, prior work has shown that the IDW2 method in California has little bias on average (0.7 ppb for NO_2,_ 0.4 µg/m^3^ for PM_2.5_, and 0.5 µg/m^3^ for PM_10_)^57,58.^ This study was also limited to one geographical region and only included Latino/Hispanic participants, which may limit generalizability. However, our focus on Latino/Hispanic women in Southern California is warranted given that this is an understudied population which experiences greater exposure to air pollution than non-Hispanic White individuals^59^. Future studies should adopt a longitudinal approach and include participants of various backgrounds from different geographic locations.

Overall, this study found that higher exposure to ambient air pollution was associated with lower HMO diversity, as well as the concentration of several HMOs. These findings hold potential importance as HMO composition is known to impact infant immunity and growth. Hence, interventions and public health policies aimed at reducing air pollution exposure may have the potential to improve infant health and development through important impacts on human breastmilk composition.

## Methods

### Participants

**Figure 4:**
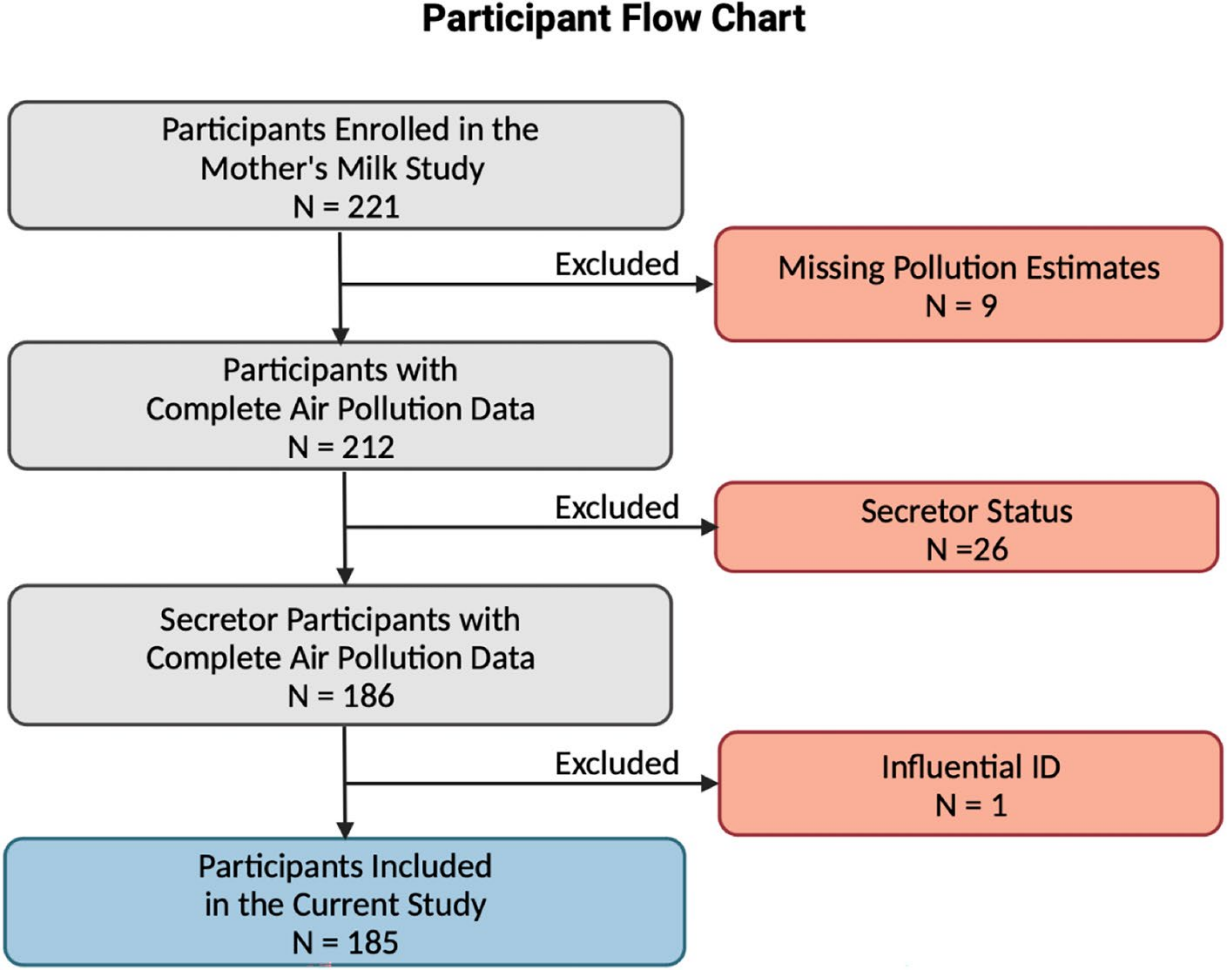
Participants included in the current analysis from the Southern California Mother’s Milk Study. Nine participants were removed due to missing air pollution estimates. Twenty-six participants were removed for having the “non-secretor” phenotype (produce little/no 2’FL). One participant was removed as they had an intermediate 2’FL value (see methods description).

The participants of this study were recruited for the Southern California Mother’s Milk Study, an ongoing longitudinal study examining human breast milk factors and the gut microbiome in a cohort of mother-infant dyads^57^. Maternal inclusion criteria include ≥ 18 years of age at delivery; healthy, term, singleton birth; enrollment by one month postpartum; self-identified Latino/Hispanic ethnicity; intention to breastfeed for at least three months postpartum; and ability to read at the 5th grade level in either English or Spanish in order to understand study procedures^60^. Mothers were excluded on the basis of medical conditions or medications which impact health, nutritional status, or metabolism, as well as use of tobacco or recreational drugs, pre-term or low birth weight, and clinically diagnosed fetal abnormalities^57^. In the current study, 185 of the 221 Mother’s Milk Study participants were included. Briefly, 9 were excluded due to missing air pollution exposure data and 23 were removed since they were classified as HMO “non-secretors” (produced under 500 nmol/mL of the HMO 2’FL). Following this, we observed one participant with intermediate 2′FL levels (559 nmol/mL, which is considered a “non-secretor" by some definitions), marking them as a potential non-secretor. This same individual had an HMO diversity > 2 SD from the mean, further suggesting they were a likely “non-secretor”. Therefore, we opted to remove this individual to maintain data integrity **(**Fig. 4**).** Additionally, we examined extreme air pollutant exposure values, which we defined as > 3 SD above the mean. Since the high exposure levels observed were plausible, we conducted thorough model diagnostics to confirm that retaining these cases did not unduly influence the analysis. The Institutional Review Boards of the University of Southern California, Children’s Hospital of Los Angeles, and the University of Colorado Boulder approved study procedures, and all research was performed in accordance with relevant guidelines and regulations. Written informed consent was obtained from participants prior to enrollment and all analyses in this study.

### Clinical measures

The clinical measures used for this study were taken at the baseline visit for participants (1-month postpartum). Maternal weight (kg) was measured using an electronic scale and standing height was measured using a stadiometer (m) to calculate maternal body mass index (BMI, kg/m^2^). Maternal pre-pregnancy weight status was categorized as having underweight (≤ 18.5 kg/m^2^), healthy weight (18.5–24.9 kg/m^2^), overweight (25.0–29.9 kg/m^2^), or obesity (≥ 30 kg/m^2^). Maternal pre-pregnancy weight, age at delivery, delivery mode, and infant sex were collected at the first clinical visit. Non-consecutive 24-h dietary recalls were performed to represent average maternal dietary intake, as well as to assess dietary information pertaining to nutrient consumption of macronutrients^61^. Socioeconomic status was measured using a modified version of the Hollingshead index^57,62^.

### Ambient air pollution exposure

Residential exposure estimates to ambient air pollutants (PM_2.5_, PM_10_, NO_2_, and O_3_) during the pregnancy period (281 days) through 1-month postpartum (30 days) were modeled for all mother-infant pairs. PM_2.5_ and PM_10_ were measured in micrograms per cubic meter (µg/m^3^) while NO_2_ was assessed in parts per billion (ppb). Residential address histories were obtained from a questionnaire during the study visit and included the prenatal period. Addresses were geocoded with the Texas A&M Geocoder. Monthly averages of pollutant exposure were then estimated using the Environmental Protection Agency (EPA) Air Quality System, which uses monitors to record hourly air quality data. Spatial interpolation of up to four monitoring stations within 50 km of participant’s homes was performed via an inverse distance-squared weighting (IDW2) algorithm. Final air pollution exposures were calculated using a weighted average of exposure during pregnancy (281 days) and 1 month postpartum (30 days).

### Human milk oligosaccharides (HMO)

Human milk was collected at 1-month postpartum (mean days after delivery: 32.47), and mothers were instructed to refrain from eating for 1 h and feeding or pumping human milk for 1.5 h before collection. Mothers were instructed to use an electric breast pump and were asked to pump the entire contents of a single breast expression to ensure collection of fore, mid, and hind milk. 20–50 mL of milk was collected, and aliquots were stored at -80 ºC until HMO analysis. HMOs were analyzed using high performance liquid chromatography after fluorescent derivatization allowing for quantification of the 19 most abundant HMOs, which represent more than 90% of the total HMO concentration, and include all structural features found in HMOs as a whole (Berger et al.^60^). HMO concentrations are reported in nmol/mL, and the sum of all HMOs in a sample was calculated as the sum of all HMOs detected in each sample. HMO-bound fucose and HMO-bound sialic acid were calculated as the sum of all sialic acid and all fucose molecules bound to HMOs in a sample, respectively (e.g., each molecule of 2′FL contains 1 molecule of fucose, and each molecule of DFLNT contains 2 molecules of fucose). HMO diversity was calculated using Simpson’s diversity, which is the reciprocal sum of the square of relative abundance for each measured HMO^42^. Additionally, as secretor status is known to impact the concentration of HMOs, only secretors (as defined by the presence of > 500 nmol/mL 2′FL HMO) were included in the analysis. Overall, 19 HMOs were identified and quantified: 2′-fucosyllactose (2′FL), 3-fucosyllactose (3FL), 3′-sialyllactose (3′SL), 6′-sialyllactose (6’SL), difucosyllactose (DFLac), difucosyllacto-*N*-hexaose (DFLNH), difucosyllacto-*N*-tetrose (DFLNT), disialyllacto-*N*-hexaose (DSLNH), disialyllacto-*N*-tetraose (DSLNT), fucodisialyllacto-*N*-hexaose (FDSLNH), fucosyllacto-*N*-hexaose (FLNH), lacto-*N*-fucopentaose (LNFP) I, LNFP II, LNFP III, lacto-*N*-hexaose (LNH), lacto-*N*-neotetraose (LNnT), lacto-*N*-tetrose (LNT), sialyl-lacto-*N*-tetraose b (LSTb), and sialyl-lacto-*N*-tetraose c (LSTc).

### Statistical analysis

**Figure 5:**
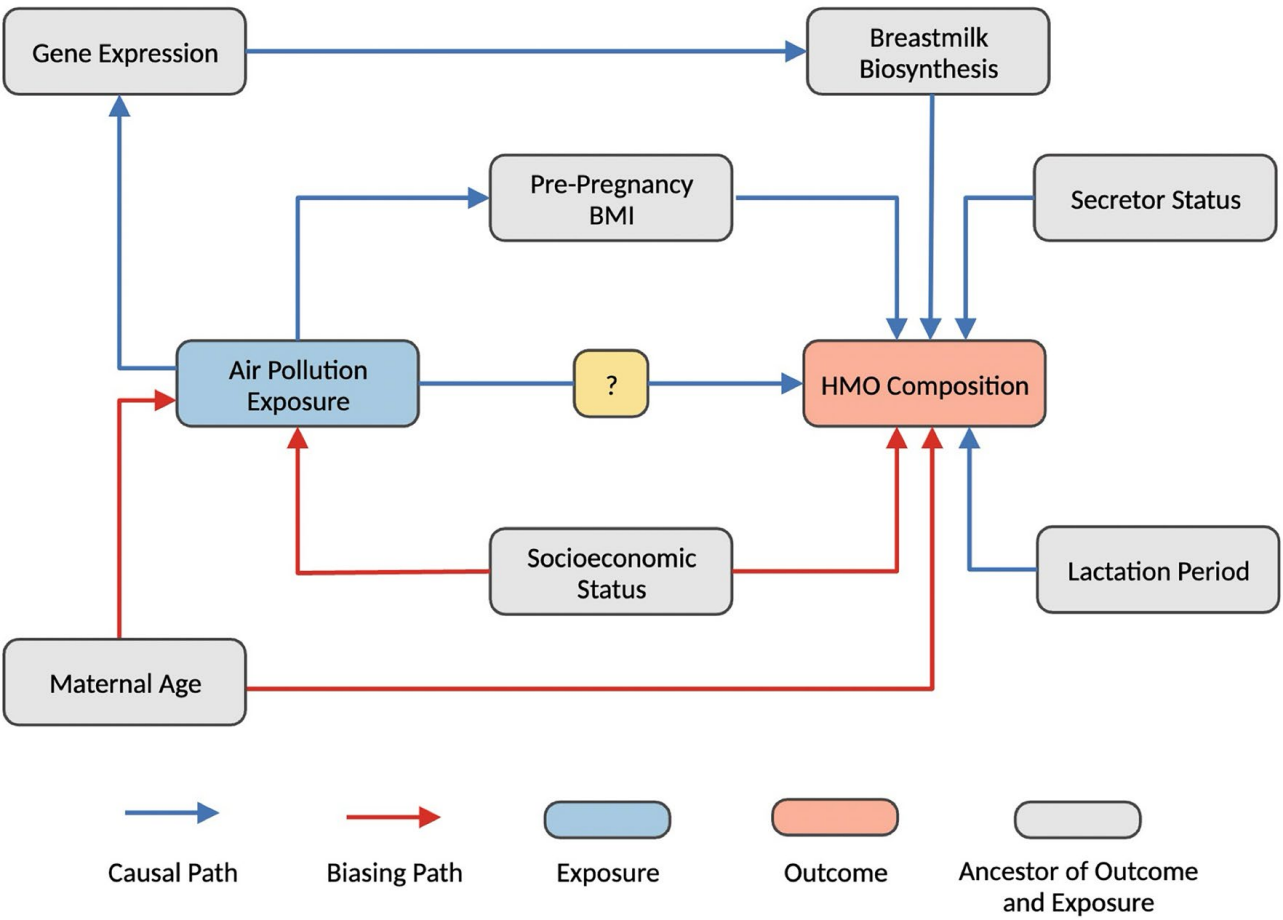
Directed acyclic graph (DAG). Factors associated with air pollution exposure and HMOs are indicated in the DAG, which was used to identify adjustment variables. Based on this DAG, both maternal age and socioeconomic status were identified as confounding variables and included in our statistical models.

Descriptive statistics were calculated using the mean and standard deviation (SD) for continuous variables and frequencies for categorical variables. Multivariable linear regression was performed to explore the relationship between exposure to air pollution and six a priori breastmilk HMOs. Since PM_2.5_ had a bimodal distribution, we also examined average differences in HMOs levels based on high and low PM_2.5_ exposure, which was based on a median split (median: 11.45 µg/m^3^, 49.19% above and 50.81% below the SD). A directed acyclic graph (DAG) was constructed based on a review of the literature (Fig. 5) to identify potentially important adjustment factors, which included socioeconomic status and maternal age. A principal component analysis (PCA) was also performed as a data reduction method. Given the directed nature of these analyses, we report unadjusted *p* values. Lastly, multivariable linear regression was used to examine the relationship among AAP and all individual HMOs. Since this analysis was exploratory, we report the raw *p* values and adjusted *p* values using the Benjamini–Hochberg procedure with an 5% false discovery rate (FDR). All effect estimates (betas) are reported for a one standard deviation increase in NO_2_ (SD: 2.23 ppb), PM_10_ (SD: 3.65 µg/${m}^{3}$), and PM_2.5_ exposure (SD: 1.02 µg/${m}^{3}$). All statistical analyses for this study were performed in R (Version 4.0.3), and a significance threshold of *p* < 0.05 was used.

### Supplementary Information

Supplementary Information.

## Data Availability

Data cannot be shared publicly because they include potentially identifying information on human subjects. The data that support the findings of this study are available upon reasonable request from the corresponding author, TLA.
