# Supplementary material for: Air pollution exposure may impact the composition of human milk oligosaccharides
Source: Sci Rep. 2024 Mar 20;14:6730. doi: 10.1038/s41598-024-57158-z (PMC10954706; doi:10.1038/s41598-024-57158-z)
Supplement: Supplementary file 1 — Supplementary Information. [file 41598_2024_57158_MOESM1_ESM.pdf]

Air pollution exposure may impact the composition of human milk oligosaccharides

Noopur C Naik[1,2], Elizabeth A Holzhausen[1], Bridget N Chalifour[1], Maria M Coffman[1], Fredrick Lurmann[3], Michael I Goran[4], Lars Bode[5], Tanya L Alderete[1]*

[1]Department of Integrative Physiology, University of Colorado Boulder, Boulder, CO, USA

[2]Cleveland Clinic Lerner College of Medicine at Case Western Reserve University College of Medicine, Cleveland, OH, USA

[3]Sonoma Technology, Inc., Petaluma, CA, USA

[4]Department of Pediatrics, Children's Hospital of Los Angeles, Los Angeles, CA, USA

[5]Department of Pediatrics, Larson-Rosenquist Foundation Mother-Milk-Infant Center of Research Excellence (MOMI CORE), Human Milk Institute (HMI), University of California, San Diego, La Jolla, CA, USA.

Supplementary Table 1. Means and Standard Deviations for HMOs

HMO Summary Measures	Mean	SD
Diversity	4.90	1.82
Sum (nmol/mL)	17124.66	1765.88
HMO-bound sialic acid (nmol/mL)	3519.67	774.71
HMO-bound fucose (nmol/mL)	14806.64	1898.41
**HMO Concentrations**		
2’FL (nmol/mL)	7062.59	3052.43
3FL (nmol/mL)	951.31	1366.82
LNnT (nmol/mL)	571.67	306.97
3’SL (nmol/mL)	560.44	521.91
DFLac (nmol/mL)	472.53	304.40
6’SL (nmol/mL)	1000.29	328.02
LNT (nmol/mL)	1088.42	610.92
LNFP I (nmol/mL)	1480.39	1186.41
LNFP II (nmol/mL)	843.40	397.66
LNFP III (nmol/mL)	60.98	36.51
LSTb (nmol/mL)	87.67	65.27
LSTc (nmol/mL)	319.04	140.44
DFLNT (nmol/mL)	1428.17	749.73
LNH (nmol/mL)	102.90	63.85
DSLNT (nmol/mL)	404.21	192.96
FLNH (nmol/mL)	161.86	114.49
DFLNH (nmol/mL)	156.87	132.26
FDSLNH (nmol/mL)	130.95	99.77
DSLNH (nmol/mL)	240.95	101.76

Supplemental Table 1. Means and standard deviations for all HMOs (summary measures and individual HMO concentrations) are given.

Supplemental Figure 1. Correlations between maternal age and body mass index with HMO in the Mother's Milk Study

	Maternal Age	PrePreg BMI	2'FL	3FL	LNnT	3'SL	DFLac	6'SL	LNT	LNFP I	LNFP II	LNTP III	LSTb	LSTc	DFLNT	LNH	DSLNT	FLNH	DFLNH	FDSLNH	DSLNH
Maternal Age	1	0.18	0.07	-0.03	-0.07	0.1	0.08	0.14	-0.01	-0.1	0.04	0.05	0.03	-0.03	-0.03	0.04	-0.02	0.04	-0.06	0.1	0.15
PrePreg BMI	0.18	1	0	0.03	-0.05	-0.09	-0.11	0.02	0.11	0.01	0.06	-0.04	0.1	-0.04	-0.04	-0.04	0.01	0.08	0	0.07	-0.04

Supplemental Figure 1. Correlation matrix between maternal age (years) and pre-pregnancy body mass index (BMI, kg/m^3) with HMOs (nmol/mL) using Pearson correlation coefficients. The color of each square, either red (negative) or blue (positive), denotes the direction of the correlation.

Supplemental Figure 2. Contribution of various HMOs to PC1 and PC2

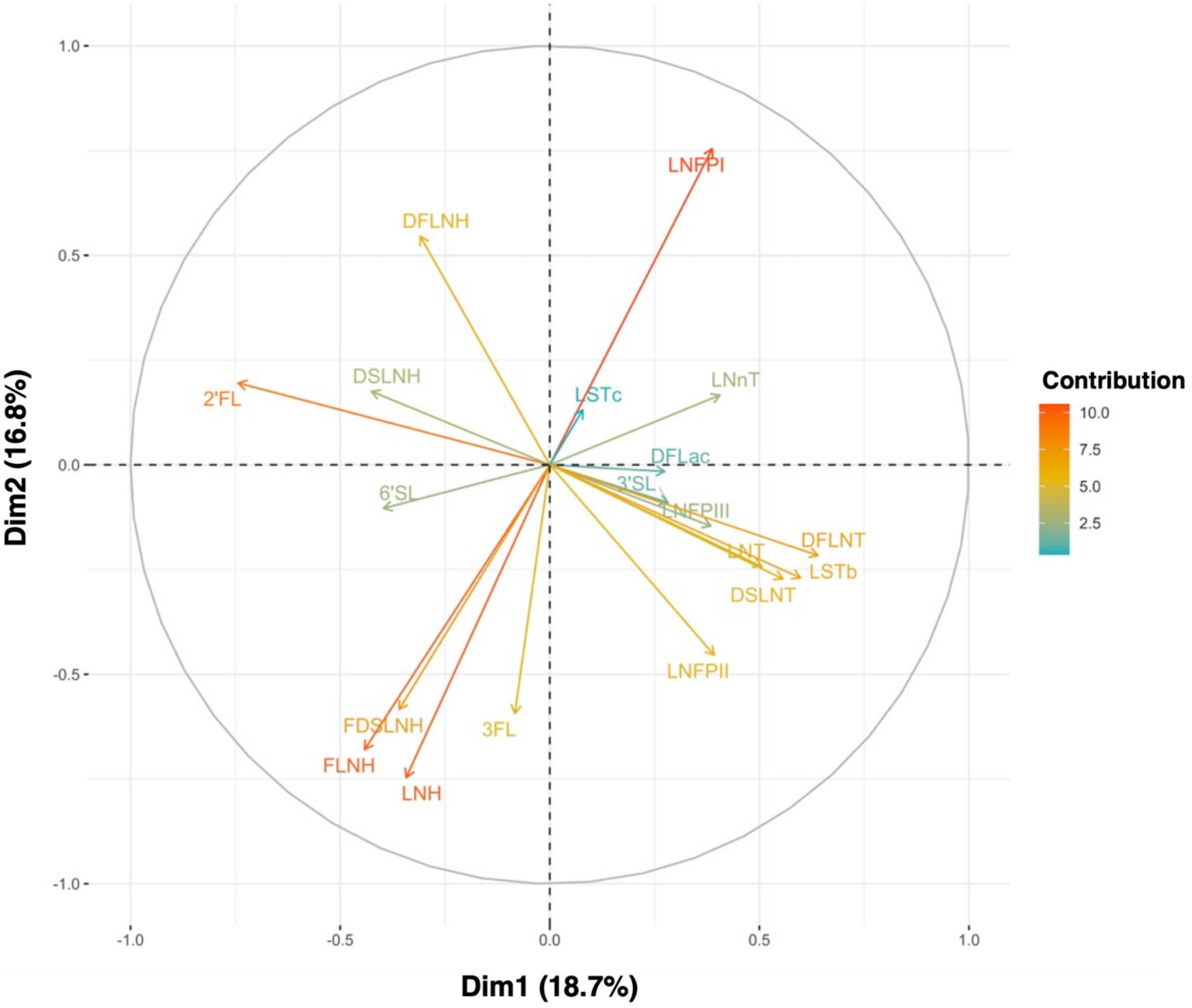

Supplemental Figure 2. Plot of the loadings of each HMO (nmol/mL) against PC 1 and 2 (labeled as Dim 1 and Dim 2). The HMOs are color coded based on their contribution to the PC. PC1 is plotted on the x-axis and PC2 is plotted on the y-axis. Together, these PCs explain approximately 35.5% of the variation in the HMO data. Variables positively correlated point to the same side of the graph and variables negatively correlated point to opposite sides. The closer an HMO is to the x-axis, the more it is defined by PC1, and the closer it is to the y-axis, the more it is defined by PC2.
